# Mechanical Properties of Biochar–Sulfur Composites

**DOI:** 10.3390/ma19030549

**Published:** 2026-01-30

**Authors:** Ewa Syguła, Monika Słupska, Maja Radziemska, Andrzej Białowiec

**Affiliations:** 1 Institute of Environmental Engineering, Warsaw University of Life Sciences, Nowoursynowska 159, 02-776 Warsaw, Poland; ewa_sygula@sggw.edu.pl; 2 Institute of Agricultural Engineering, Wroclaw University of Environmental and Life Sciences, 37 Chełmonskiego Str., 51-630 Wroclaw, Poland; monika.slupska@upwr.edu.pl; 3 Department of Applied Bioeconomy, Wroclaw University of Environmental and Life Sciences, 37a Chełmonskiego Str., 51-630 Wroclaw, Poland; andrzej.bialowiec@upwr.edu.pl; 4 Lukasiewicz Research Network—PORT Polish Center for Technology Development, Stablowicka 147 Str., 54-066 Wroclaw, Poland

**Keywords:** sulfur, biochar, mechanical properties, composite structure

## Abstract

The study examines the mechanical strength of sulfur–biochar composites (SBCs), an underexplored area with potential for developing robust materials. Sulfur production, primarily from specialized extraction and waste generation in petroleum refining, yields about 70 million tons annually, necessitating efficient waste management. SBCs were produced using waste-derived biochar and elemental sulfur at varying sulfur contents (60–80%) and employing two fabrication methods: a muffle furnace and an electric burner. The mechanical performance of the composites was evaluated through strength and displacement measurements, with particular emphasis on the influence of processing method and sulfur content. The results demonstrate that both sulfur content and fabrication method significantly affect the mechanical behavior of SBCs. An increase in sulfur content led to a systematic improvement in ultimate strength for all samples. However, composites produced using the electric burner exhibited markedly higher ultimate forces and lower displacements compared to those fabricated in the muffle furnace, indicating superior strength and reduced brittleness. The enhanced performance is attributed to improved sulfur distribution and more effective infiltration of liquid sulfur into the porous biochar structure. These findings confirm the synergistic effect of combining sulfur with biochar and highlight the critical role of processing conditions in developing mechanically robust sulfur–biochar composites suitable for sustainable material applications.

## 1. Introduction

### 1.1. Application of Waste Sulfur in Materials

#### 1.1.1. Sulfur Production

Sulfur is extracted through two sectors: specialized and attendant. The first sector relies on extracting native sulfur from primary deposits, primarily found in Iraq, the United States, Chile, Mexico, and Poland [1]. On the other hand, the second sector involves the production of waste sulfur during the purification and desulfurization processes of petroleum products [2]. The element is recovered through the Claus process, in which hydrogen sulfide decomposes into elemental sulfur [3]. Annual sulfur production is estimated to reach 70 million tons [4], with the first sector responsible for only 10% of extraction, resulting in the closure of an increasing number of mines [5]. The world is struggling to manage the enormous amounts of sulfur waste generated by the petrochemical industry. According to data from the U.S. Geological Survey, sulfur recovery from petrochemical processes increased by 19% from January to March 2022 compared to the previous year [6]. The United Arab Emirates is the largest sulfur exporter, accounting for over 95% of its production, representing 25% of global sulfur production, and it is predicted to double in the next 10–15 years [7]. However, the demand for sulfur will not increase at the same pace, which is why finding methods to manage this element is crucial. In the past, sulfur was primarily used as a medicine and was also utilized in the production of gunpowder and bleaching agents [6]. Currently, due to its numerous physical, chemical, and mechanical properties, sulfur offers a wide range of applications, including tire vulcanization, is an essential component in artificial fertilizers, and is increasingly used in the construction sector (sulfur-based concrete) [8], as well as replaces lithium in lithium-ion battery cathodes [4,9] and is used for water electrolysis for hydrogen production [10].

#### 1.1.2. Mechanical Properties of Sulfur

Sulfur exhibits several allotropic forms: monoclinic, rhombic, and plastic, each varying in their state of matter and viscosity depending on prevailing temperatures [2]. Additionally, the mechanical properties of sulfur are influenced by thermal conditions and temperature. Its compressive strength is measured at 22 MPa, while its tensile strength is entirely dependent on cooling rate and temperature [6]. Faster cooling and higher target temperatures yield better mechanical strength parameters. Utilizing sulfur as an aggregate binder, concrete can achieve compressive strengths of up to 48 MPa, marking a 37% increase compared to traditional Portland cement (PC) concrete [11]. This improvement stems from the low porosity of sulfur-modified concrete in the absence of water. In traditional concrete, excessive water needed for cement hydration leads to void formation, thereby reducing mechanical strength. Moreover, studies have demonstrated that the addition of sulfur enhances tensile strength by 10% compared to PC. Numerous investigations corroborate the positive impact of sulfur addition on concrete’s mechanical strength [9,11]. Furthermore, sulfur enhances concrete’s resistance to acidic and saline environments, attributed to its lower calcium oxide content and higher aluminum oxide content relative to PC [10]. Sulfur properties are also harnessed in conjunction with polymers through inverse vulcanization. By adjusting sulfur and divinylbenzene and 1,3-diisopropenylbenzene ratios appropriately, materials with desired characteristics can be obtained. Samples with higher sulfur concentrations exhibit amorphous properties with low flexibility and high shape retention, while those with lower concentrations display low viscosity and high fluidity [12]. Incorporating sulfur into composites allows manipulation of their mechanical properties, leveraging sulfur’s unique array of physicochemical properties [13]. In parallel with sulfur-based modification, modern concrete technology increasingly employs fiber reinforcement, including polypropylene (PP) fibers, to improve crack resistance and post-failure behavior. Recent studies have demonstrated that recycled PP fibers can effectively enhance the mechanical performance of cementitious materials, contributing to sustainability-oriented construction solutions. The potential combination of sulfur-modified concrete with polymer fiber reinforcement represents a promising, yet largely unexplored, research direction [14].

#### 1.1.3. Usage of Waste Sulfur in Materials

Currently, sulfur finds application in various sectors, with the largest being the fertilizer industry. Biologically, sulfur serves as an essential element for plant organisms, offering direct nutritional value and enhancing the effectiveness of other necessary elements [15]. Studies have demonstrated that growth stimulators containing sulfur are readily absorbed by wheat seeds, hastening germination and ensuring early plant yield. Incorporating elemental sulfur into fertilizers can boost plant yield by up to 50% while also reducing soil pH and redox potential. In cases of arsenic-contaminated soil, sulfur diminishes its value by over 40% [16]. An important consideration in the use of sulfur-containing fertilizers is the form in which it is applied. Fertilizers containing sulfur in dry or solution form contribute to increased yields due to the nanostructure form of the element, facilitating its penetration into plants. Utilizing both forms of fertilizer positively impacts morphological parameters, plant height, and the vegetative period’s development [17]. Another sector where sulfur finds utility is the construction industry. Sulfur serves as a binder in the production of concrete, referred to as sulfur–biochar composites (SBCs). Unlike traditional PC, SBC production does not necessitate the use of water or cement, which are major contributors to high CO_2_ emissions [2]. Consequently, SBC yields material with twice the mechanical strength and reduced environmental impact. While the use of sulfur in construction materials offers numerous benefits, its application is limited due to its physical properties (melting point above 140 °C) [18]. Potential applications of SBCs include constructing dams, bridges, irrigation channels, or producing sewage pipes. Sulfur is commonly employed as a raw material for specialized polymers. The vulcanization process aims to produce durable tires. Sulfur’s inclusion in this process enhances the physical properties of natural rubber. Additionally, the polymerization of sulfur and alkenes yields a chemical substance used for pollution control, monitoring, and remediation [19]. Polysulfides formed during the inverse vulcanization process using elemental sulfur and limonene compound from lemon peel can effectively remove mercury pollutants from water. Adding sulfur to polymers enhances their tensile strength and Young’s modulus [20]. The increasing market demand is driving the exploration of solutions offering higher energy density. Lithium–sulfur batteries, boasting a high energy efficiency of 26 kWh∙kg^−1^ and a large depth of discharge, present a promising avenue. However, their main drawback lies in the low electrical conductivity of the cathode and poor cycle life due to the dissolution of polysulfides in the electrolyte [21,22]. Similarly, sulfur may be used for porous nickel–sulfur electrodes, which may be applied for hydrogen generation from water electrolysis, especially with brackish water [10].

### 1.2. Application of Biochar in Materials

#### 1.2.1. Biochar Production

Biochar is a product of the thermal decomposition of biomass under oxygen-free conditions. Mostly, it is obtained through pyrolysis, which can be categorized into slow or fast pyrolysis depending on specified parameters of time and temperature. Torrefaction, gasification, or hydrothermal carbonization (HTC) may also be a source of biochar. Pyrolysis occurs at temperatures ranging from 200 to 900 °C, during which cellulose, hemicellulose, and lignin undergo depolymerization and fragmentation reactions [23]. By limiting oxygen access, carbon combustion is prevented, leading to the production of pyrolytic gas, pyrolytic oil, and biochar [24]. The quantity and quality of the products obtained depend on the appropriate selection of process parameters and the feedstock used. Gasification of biomass primarily yields syngas, with biochar accounting for 10% of the feedstock [25]. Studies indicate that the amount of biochar produced decreases with increasing temperature [26]. However, the ash content, pH, porosity, and specific surface area of the resulting carbonization product increase [27,28]. Biochar produced at 750 °C exhibited a specific surface area of 540.36 m^2^∙g^−1^, while biochar from a process set at 350 °C had a specific surface area of 1.44 m^2^∙g^−1^ [29]. Both temperature and the duration of the biomass process influence the characteristics of biochar. Increasing the process time has been found to decrease porosity and specific surface area [30].

#### 1.2.2. Mechanical Properties of Biochar

The type of biomass used in the pyrolysis process significantly influences the physicochemical and mechanical properties of the resulting biochar. Researchers have demonstrated that biochar produced from sugarcane bagasse or rice husk exhibits a high nutrient content and electrical conductivity [31]. Utilizing carrot peels as raw material in the pyrolysis process yields carbonization with high adsorption potential. It has been shown that biochar derived from plant biomass has a high carbon content [32]. The reactor employed in the process can also impact the mechanical properties of biochar. Comparing the carbonization results from an HTC reactor (300 °C), a fixed bed horizontal tube furnace reactor (300 °C and 900 °C), and a fixed bed vertical batch reactor (300 °C and 900 °C), differences in Young’s modulus hardness were observed. All biochars set at a temperature of 300 °C in different reactors displayed significant differences in Young’s modulus hardness results. Biochar obtained from an HTC reactor exhibited the lowest result, at 143.1 MPa. The presence of volatile tars and trapped water after the process enhances the plasticity of biochar. In the vertical batch reactor, the temperature increase resulted in higher modulus yields, ranging from 1452 MPa to 3019 MPa. This phenomenon is because, at lower temperatures, the breakdown of cellulose results in a viscoplastic biochar. Interestingly, the trend observed in the horizontal reactor was the opposite. The carbonization process set at a higher temperature of 900 °C yielded a hard result of 1419 MPa, while the process set at 300 °C resulted in 3867 MPa. The type of reactor, with different N_2_ flow rates, influenced this parameter [33]. Therefore, it is crucial to select appropriate process parameters and the reactor used. In other studies, it has been found that the addition of biochar to polylactic acid (PLA) increases the Young’s modulus hardness of the resulting composite. This is attributed to the strong intermolecular interaction between biochar and PLA, where biochar molecules restrict the mobility of the PLA chain [34]. Additionally, it has been observed that the addition of biochar decreases the tensile strength of the composite, indicating an inability to transfer stress as the cause [35].

#### 1.2.3. Usage of Waste Biochar in Materials

New applications of biochar are the subject of extensive research. Scientists unanimously agree on one aspect: that the feedstock and process methodology significantly impact the quality of the final product. Particularly, the mechanical and thermal properties, along with the sorption capabilities for various pollutants, are modified by the process [36,37,38]. Biochar is an environmentally friendly product and relatively inexpensive to obtain, with exceptional characteristics such as high adsorption capacity, specific surface area, microporosity, and ion exchange capacity. Manipulating the pyrolysis process allows the production of biochar with desired properties for various applications. Studies have demonstrated that using biochar as a soil amendment improves soil microbial activity by providing essential nutrients, neutralizing soil acidity, and positively influencing physicochemical properties such as pH, CEC, and water retention capacity [39,40]. Biochar can effectively absorb heavy metal ions present in soil and organic pollutants [41]. Due to its large specific surface area, hydrophobic properties, and porosity, biochar is successfully applied as an adsorbent for many pollutants in aquatic environments [42]. Heavy metal pollutants such as lead, zinc, copper, and cadmium can be efficiently adsorbed from water, and by selecting the appropriate biomass, their precipitation is also possible [43]. Research has also been conducted on the removal of nanoplastics from contaminated water. Biochar produced at higher temperatures with a specific surface area of 540.36 m^2^∙g^−1^ removed up to 99% of pollutants [29]. The main research directions for biochar are its application in soil and water remediation, as mentioned earlier. However, more studies explore the possibility of utilizing biochar as a filler in composites and plastics. Scientists, as pioneers in investigating this idea, have proposed applying biochar in wood polymer composites (WPCs) and studying its properties. The hydrophobic nature of biochar combined with WPC reduces moisture absorption, thereby enhancing the composite’s resistance to decay. Once again, it has been confirmed that the porous structure of biochar translates into improved properties. The more pores, the better the polymer can infiltrate, creating a mechanical interlock/scaffold [44]. Biochars characterized by a large specific surface area and high carbon content (up to 82%) exhibit high flexural and tensile strength, primarily due to the high process temperature. A sample with high ash content but low carbon content (up to 50%) and small specific surface area showed the highest strength modulus and the highest tensile strength. High electrical conductivity of the biochar enables its application for electrode production for water electrolysis and hydrogen production [45]. This publication is one of many supporting the thesis that biochar positively influences the mechanical properties of composites [46]. However, there is a study that presents the opposite findings. It compared pure PLA with PLA incorporating biochar, resulting in decreased flexural strength and impact strength. Although the authors confirmed the antithesis, they noted that sample preparation procedures may have influenced the results [47]. However, numerous similar applications of sulfur and biochar: agriculture, construction, batteries, water electrolysis, and many more, induce the concept of the synergistic combination of these two materials, leading to the strengthening of the desired properties and the mitigation of the weaknesses of both materials.

### 1.3. Current Research on Biochar–Sulfur Composite

Biochar–sulfur composites can also find application as soil amendments. The impacts of synthetic fertilizer and sulfur-enriched biochar (SulfaChar) on soybean and maize cultivation were compared. The biochar composite was produced by exposing it to biogas emissions from a landfill, where it adsorbed significant amounts of hydrogen sulfide present in the biogas. Improved nutrient uptake by maize plants was observed when SBC was applied. The addition of the composite increased the uptake of macronutrients such as nitrogen, phosphorus, potassium, and calcium and micronutrients, including zinc, manganese, and boron, by several percentage points compared to synthetic fertilizer. However, for soybeans, the addition of SulfaChar did not result in better results compared to fertilizer [48]. The authors were the first to confirm that hydrogen sulfide present in the composite contributed to plant growth, suggesting the use of SBC as a fertilizer. Biochar functionalized by sulfur has been successfully applied for soil and water remediation for efficient removal of metal ions, antibiotics, and dyes [49]. BSC may also be used as a bacteriocide [50] and for biogas production enhancement [51].

Previous studies on SBC have primarily focused on lithium–sulfur batteries. Lithium–sulfur batteries (Li-S) are relatively cheap compared to traditional lithium-ion batteries due to the abundance and surplus of sulfur, and they are more environmentally friendly as they do not contain toxic and heavy metals. Li-S batteries possess a high theoretical specific capacity and theoretical specific energy, but they exhibit low electrical conductivity [52]. For this reason, sulfur is often combined with materials that have high electrical conductivity, such as biochar [53,54]. Porous biochar, characterized by its large specific surface area, provides the necessary volume space for sulfur conversion to Li_2_S_2_ and Li_2_S [46]. Studies have been conducted using a core–shell composite material consisting of a sulfonated polyaniline shell and a bamboo-derived porous carbon core loaded with sulfur as the cathode in Li-S batteries. The use of this composite material resulted in a high discharge capacity of 1484 mAh∙g^−1^ at 0.1 °C, high efficiency, and cyclic stability with a capacity of 853 mAh∙g^−1^ maintained after 100 cycles at 0.1 °C [46,48]. Also, new trends in the application of sulfur-doped biochars for water electrolysis are under development [13].

The use of biochar and sulfur as fillers/binders in composites is a practice under development. Biochar, primarily derived from waste materials, is an environmentally friendly product, while sulfur is readily available and inexpensive due to its role in the petrochemical industry. Considering the development of new SBCs and their various emerging applications, an extremely important aspect is the durability of the composites produced.

### 1.4. Aim of Study

This study investigates the mechanical strength of SBC, an underexplored research area with potential for developing durable and sustainable materials. SBCs were produced using different sulfur contents and fabrication methods to evaluate the influence of processing conditions on strength and deformation behavior. Mechanical strength testing was conducted to identify the key factors governing composite performance and to assess potential application areas for the developed materials. It was hypothesized that both sulfur content and fabrication method significantly influence the mechanical strength and deformation behavior of sulfur–biochar composites.

## 2. Materials and Methods

### 2.1. Materials

#### 2.1.1. Biochar

Apple wood chips (Browin, Lodz, Poland) were utilized to produce SBC. The preparation of apple chip biochar was conducted in a muffle furnace (SNOL, 8.1/1100, Utena, Lithuania) at a temperature of 500 °C with a holding time of 1 h. The temperature ramp-up was 10 °C∙min^−1^. The process was performed in the absence of oxygen due to the application of CO_2_, with the flow rate 0.6 mL∙min^−1^. Subsequently, the biochar was ground to a mesh size of 1 mm to match the form of the sulfur for later use [8].

#### 2.1.2. Sulfur

The crystalline sulfur was supplied by the ORLEN Poland SA group (Płock, Poland). Initially, the sulfur existed as a single agglomerate, which was subsequently crushed and ground in a laboratory mortar. The resulting sulfur was in powder form, with a mesh size of 1.0 mm, facilitating its mixing with biochar while ensuring high homogeneity [10].

### 2.2. Methods

#### 2.2.1. Production of SBC

The ground test materials underwent additional sifting through a 1 mm gradation sieve to remove any larger particles. While composite materials are typically produced using high-pressure injection molding machines, the authors of this work tested whether the desired effect could be achieved using atmospheric pressure melting. The production of two types of SBCs in a muffle furnace and on an electric burner was proposed. Two different heating approaches were applied to produce SBCs to evaluate the influence of fabrication conditions on mechanical performance. The muffle furnace provides uniform, indirect heating with relatively slow heat transfer, while the electric burner enables direct and localized heating, resulting in faster sulfur melting and different solidification conditions. Four mixtures of biochar and sulfur were proposed: SBC_60 (40% biochar, 60% sulfur), SBC_65 (35% biochar, 65% sulfur), SBC_75 (25% biochar, 75% sulfur), and SBC_80 (20% biochar, 80% sulfur) as our previous study revealed that the SBCs with higher contents of biochar were not durable [51].

Initially, the four proposed mixtures were prepared in a muffle furnace (SNOL, 8.1/1100, Utena, Lithuania). The test material was placed in silicone molds, with the side wall of the mold at right angles to its base, to create samples with a base-to-height ratio of 1 to 1.5. Making the SBCs in the silicone mold facilitated easy removal of the samples. The premeasured proportions of mixed biochar and sulfur were then placed in the silicone mold and placed inside the muffle furnace. The process temperature was set at 140 °C with a process time of 2 h.

**Figure 1:**
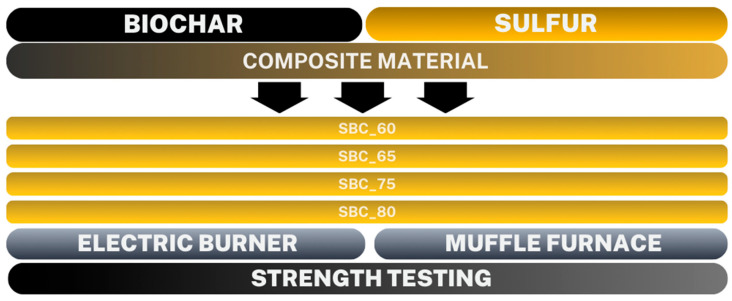
Diagram of individual SBC mixtures of the appropriate proportion with demonstration of analyses.

The second type of SBC was produced with the application of a laboratory electric burner (CAT H3.1, Ballrechten-Dottingen, Germany). Initially, the sulfur was melted at a controlled temperature of 140 °C in a thermal test chamber (KBC-100WL WAMED, Warsaw, Poland) until liquefaction. Then, the biochar was heated on an electric burner to 140 °C, with temperature control conducted using a pyrometer (Hendi 400, Rhenen, The Netherlands). Liquid sulfur was added to the heated vessel until it reached a temperature of 140 °C. The biochar was heated to homogenize the temperature of the two materials to maintain a constant mixture temperature of 140 °C throughout the process. Failure to heat the biochar would result in a lower sulfur temperature during mixing. After mixing the materials on the burner, the SBC sample was placed into the thermal test chamber for 10 min to solidify the material. The procedure for producing SBC on the burner was conducted based on the methodology for forming sulfur concrete according to Gulzar et al. [8]. The scheme of the tests conducted is shown in Figure 1, and eight mixtures of biochar and sulfur were analyzed for strength analysis.

#### 2.2.2. Structure Observation and Image Analysis

The structure of the produced SBC was examined using a Delta Optical Smart 5.0 optical microscope (Delta Optical, Mińsk Mazowiecki, Poland) at a magnification of ×100. The homogeneity and surface features of the composites were documented using a 5 MP integrated digital camera (Delta Optical DLT-Cam 5MP, Delta Optical, Mińsk Mazowiecki, Poland). The acquired images of SBC surfaces were saved in *.jpg format and further analyzed using ImageJ software (Version 1.54p).

Image processing and fractal analysis were performed using ImageJ software (Version 1.54p, NIH, Bethesda, MD, USA). Prior to analysis, the images were converted to 8-bit grayscale format and binarized using a global threshold to obtain binary images with pixel intensity values of 0 and 255. This preprocessing step was required to meet the input criteria of the fractal analysis tool. The fractal dimension was determined using the box-counting method implemented in the Fractal Box Counting plugin available in ImageJ [55]. The plugin calculates the fractal dimension by counting the number of boxes of decreasing size required to cover the binary image. All analyses were performed on the entire image area using identical processing parameters to ensure consistency and comparability between samples [56].

#### 2.2.3. Material Strength Testing

The strength properties of the biochar samples were tested in the compression test using a testing machine (Instron, Norwood, MA, USA). The samples were prepared in the shape of cylinders with a diameter of 57 mm and a height of 30 mm. In total, 24 samples were tested, 3 repetitions for each sulfur content ratio and method of SBC production [49]. During the compression test, data on force and displacement were collected. Based on the data, the ultimate strength of each sample was determined.

## 3. Results and Discussion

**Figure 2:**
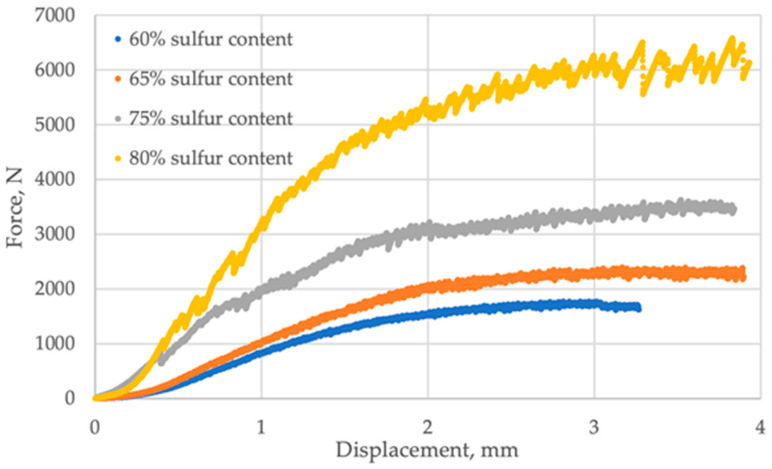
Strength properties of SBC after thermal treatment in a muffle furnace.

SBCs were produced in triplicate batches, utilizing both a muffle furnace and an electric burner. Four distinct ratios of sulfur and biochar were selected, yielding a total of twenty-four samples. In Figure 2, a representative test sample from each group is displayed. The initial observation reveals significant structural disparities between the samples fabricated in the muffle furnace and those in the electric heater. Before conducting the strength test, each sample was meticulously prepared to ensure that the ratio between the height and the diameter of the base fell within the range of 1 to 1.5. Additionally, sulfur is much more visible in the case of the SBC produced with the burner application, showing the higher level of distribution (Figure 2). Moreover, the determined particle size of sulfur showed that that of the SBC produced in the furnace was in a higher range than that in SBC from the burner (Table 1). This may be attributed to the liquid sulfur better penetrating the voids between biochar particles.

The fractal dimension (Df) values obtained for SBC produced using different sulfur contents and fabrication methods are presented in Table 2. For both production routes, an increase in sulfur content resulted in a systematic increase in fractal dimension, indicating higher surface structural complexity. For samples produced in the muffle furnace, the fractal dimension increased gradually from 1.3741 for SBC_60 to 1.5253 for SBC_80. This trend suggests a progressive increase in surface heterogeneity with increasing sulfur content. However, the absolute Df values remained relatively moderate, reflecting a less complex surface structure. In contrast, samples produced using the electric burner exhibited generally higher fractal dimension values, particularly at higher sulfur contents. The Df increased from 1.3408 for SBC_60 to 1.6198 for SBC_80. The most pronounced increase was observed between SBC_65 and SBC_75, indicating a significant change in surface structural complexity associated with higher sulfur incorporation under electric burner processing conditions. The higher fractal dimension values observed for electric-burner-treated samples suggest a more complex and irregular surface morphology compared to samples produced in the muffle furnace. This difference may be attributed to variations in sulfur melting, distribution, and solidification behavior resulting from the distinct thermal conditions of the two fabrication methods. Overall, the fractal dimension analysis confirms that both sulfur content and production method influence the image-based structural complexity of SBC surfaces. Similar relationships between processing-induced structural features of biochar-containing materials and their mechanical performance have been reported in previous studies, where image-based or microstructural observations were linked to compressive strength without direct microvoid quantification [57,58]. In this context, the fractal dimension applied in the present study serves as a qualitative descriptor of surface structural complexity rather than a direct measure of porosity.

**Figure 3:**
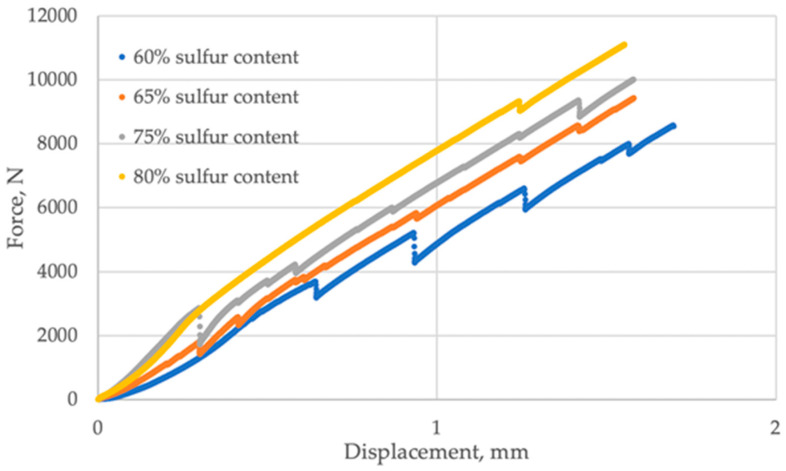
Strength properties of SBC after thermal treatment on an electric burner.

The average results of the strength properties of the SBC samples are presented in Figure 2 and Figure 3. It was observed that the method of composite preparation highly influenced the quality of the biochar. The mixtures prepared in the muffle furnace were characterized by lower strength properties values than SBC made using the electric burner. It can be explained by the density of the material influenced by its porosity, resulting from the air content and bond strengths between carbon molecules. The study showed that, with the increase in the sulfur content in biochar, the ultimate strength also increases. For furnace treatment, the ultimate strength of the sample with a sulfur content of 60% scored about 2000 N, while the sample with 80% sulfur content achieved an about 3.5 times higher value of ultimate strength. For electric burner treatment, the ultimate strength of SBC, with a share of sulfur from 60 to 80%, increased from 8000 to 11,000 N, respectively.

The samples subjected to furnace treatment exhibited increased brittleness, with the propagation of surface cracking initiating at 500 N and remaining relatively constant thereafter. This phenomenon can be attributed to higher porosity and weaker bond strengths between carbon molecules in the furnace-treated samples.

Table 2 provides data on the ultimate force and displacement for SBC samples treated under different conditions: in a muffle furnace and on an electric burner, varying the sulfur content. For samples treated in the muffle furnace, at 60% sulfur content, the ultimate force was 1770 N with a displacement of 3.27 mm. At 65% sulfur content, the ultimate force increased to 2393 N while the displacement increased to 3.90 mm. With 75% sulfur content, the ultimate force further increased to 3623 N with a displacement of 3.84 mm. At the highest sulfur content of 80%, the ultimate force substantially rose to 6577 N with a displacement of 3.93 mm. For samples treated on the electric burner, at 60% sulfur content, the ultimate force significantly increased to 8570.36 N, while the displacement decreased to 1.70 mm. With 65% sulfur content, the ultimate force further rose to 9417 N, with a slight decrease in displacement to 1.58 mm. At 75% sulfur content, the ultimate force continued to increase to 10,002 N, with displacement remaining at 1.58 mm. Finally, at 80% sulfur content, the ultimate force peaked at 11,093 N, while the displacement decreased slightly to 1.55 mm. These results suggest that samples treated on the electric burner exhibit significantly higher ultimate forces and lower displacements compared to those treated in the muffle furnace, indicating superior strength characteristics. Additionally, as the sulfur content increases, the ultimate force generally increases across both treatment methods, albeit at different rates.

**Figure 4:**
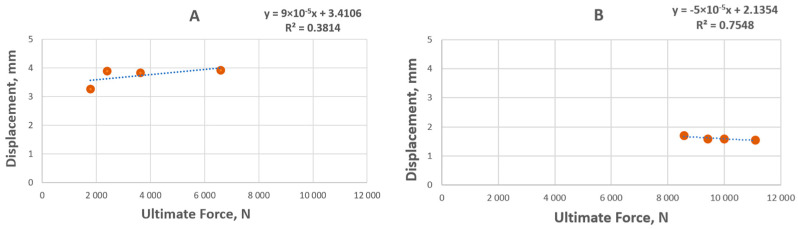
Relationship between ultimate force and displacement of the SBC for the furnace (**A**) and electric burner (**B**). The dotted lines represent linear regression fits; the corresponding regression equations and coefficients of determination (R^2^) are shown in the plots.

Additional correlative analyses revealed that both SBC types behave differently during the performance of the force. Furnace SBC displacement increases with the increase in the force; however, in the case of the electric burner SBC, this phenomenon is the opposite (Figure 4). This may indicate that the method of SBC production significantly impacts the mechanical properties of the produced composites. When the liquid sulfur is mixed with the hot biochar, it better fills the voids and porous structure of the biochar, which is in alignment with the results of sulfur particle size (Table 3).

## 4. Conclusions

The preliminary tests on sulfur–biochar composites indicate a promising avenue for developing materials with commendable mechanical properties, sans the need for high-pressure injection molding machinery. An escalation in sulfur content leads to improved composite strength, highlighting the efficacy of blending sulfur with biochar to counteract the typical brittleness associated with such composites.

Significant differences emerged between samples treated with an electric burner versus those from a muffle furnace. Notably, samples treated with the electric burner exhibited a wider range of strength, with the SBC_80 variant reaching an impressive 11 kN. The correlation between biochar strength and sulfur content is apparent from the data, with biochars treated in an electric burner demonstrating notably higher durability (lower displacement) compared to those from muffle furnace treatment. Analysis of the ultimate force and displacement data suggests that samples treated in the muffle furnace experienced a notable strength increase with rising sulfur content. Conversely, samples treated with the electric burner displayed even greater increases in ultimate force, alongside lower displacements, further highlighting the superior strength characteristics of the electric-burner-treated samples. This may be related to better sulfur distribution and filling of the pores and voids between biochar particles.

The study further reveals a direct correlation between sulfur content in biochar and ultimate strength, with consistent increases observed across both furnace and electric burner treatments.

## Figures and Tables

**Table 1:** Sulfur particles’ size concerning the SBC production method and the content of sulfur.

Thermal Treatment	Sulfur Content, %	Average Size of Sulfur Particles, Pixels
Muffle Furnace	60	22.3
	65	26.1
	75	36.2
	80	66.1
Electric Burner	60	20.9
	65	24.4
	75	47.1
	80	54.8

**Table 2:** Surface images of sulfur–biochar composites (SBCs) produced using a muffle furnace and an electric burner at different sulfur contents, together with the corresponding fractal dimension (Df) values.

	SBC_60	SBC_65	SBC_75	SBC_80
D_f_	1.3741	1.3918	1.4899	1.5253
Muffle Furnace	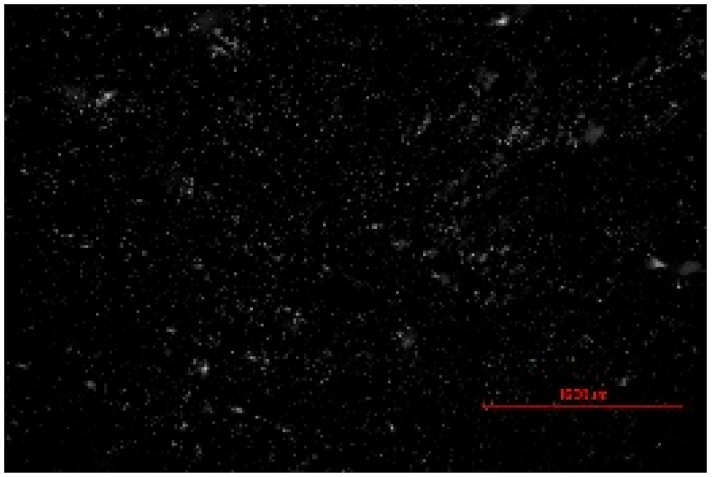	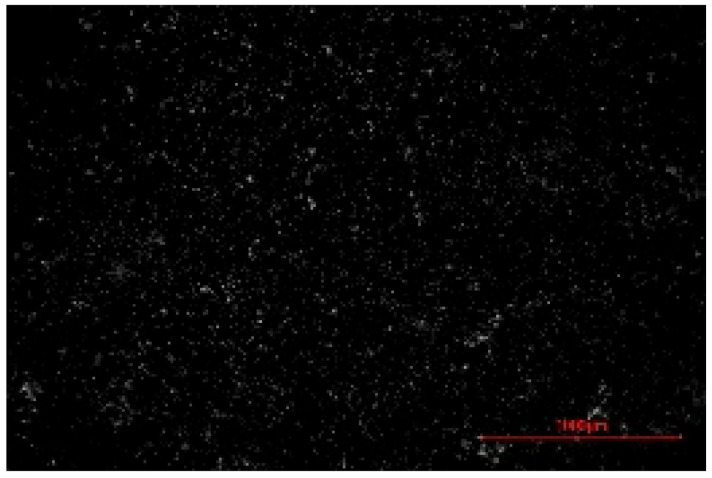	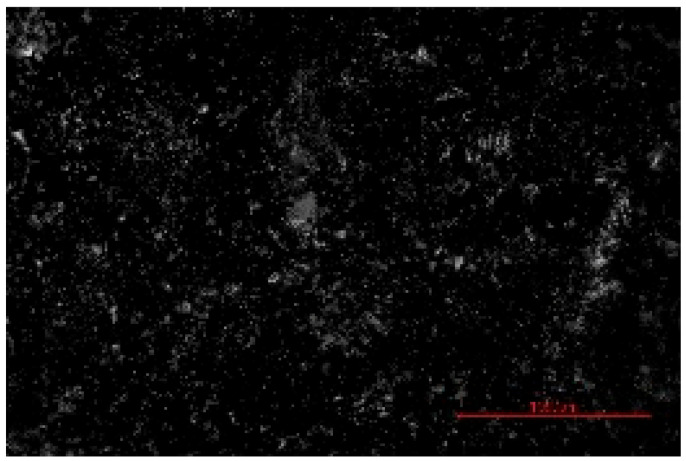	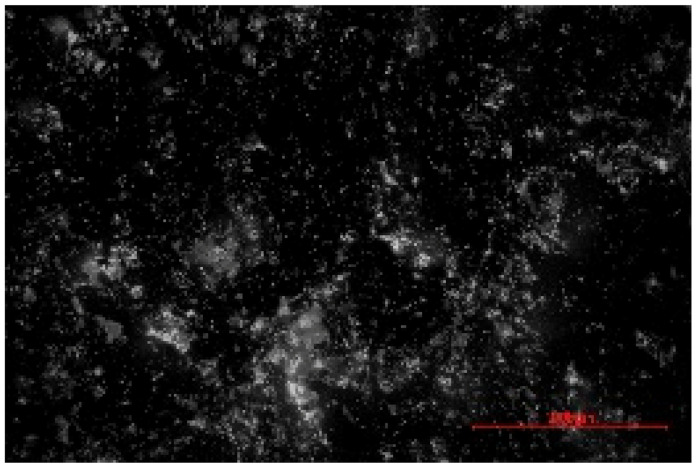
D_f_	1.3408	1.4273	1.6189	1.6198
Electric Burner	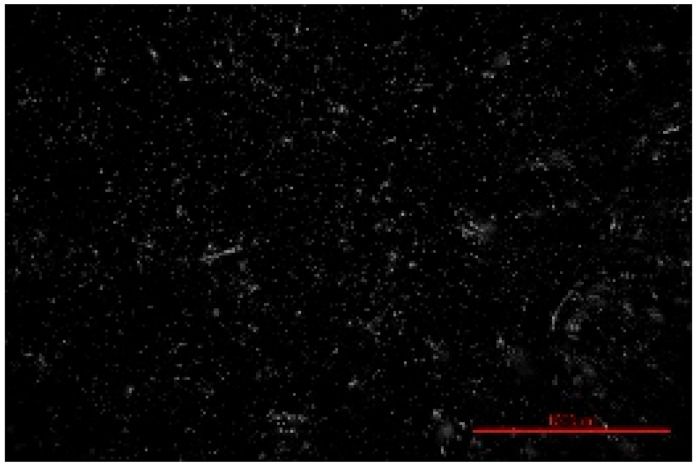	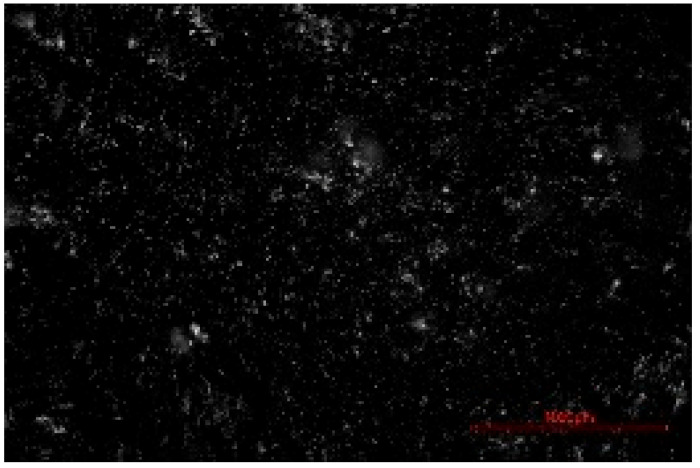	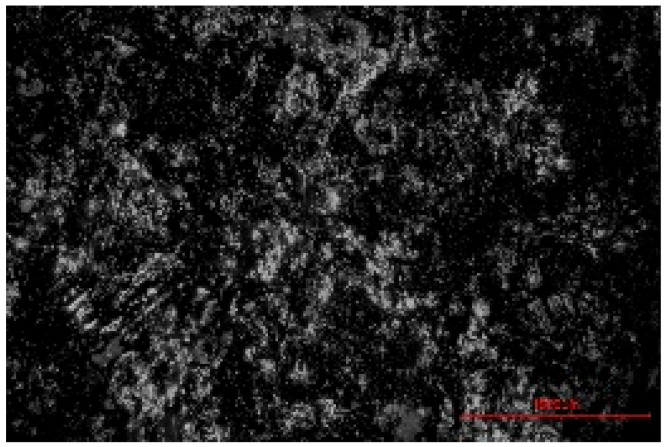	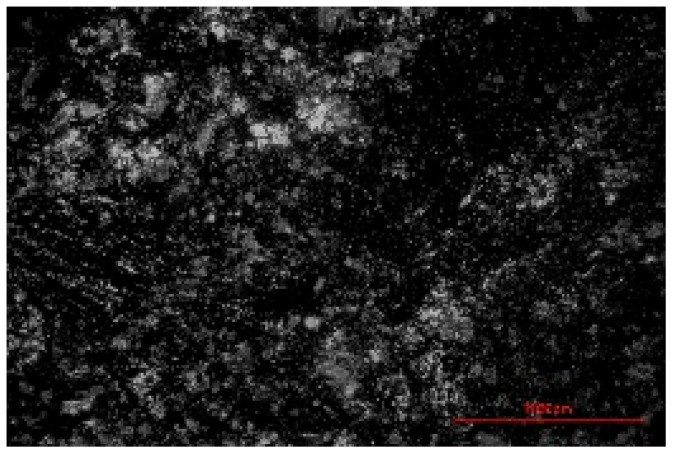

All images were acquired at the same magnification. The scale bar in each image corresponds to 1000 µm. D_f_ denotes the fractal dimension calculated from the binarized images.

**Table 3:** Summary of ultimate strength results.

Thermal Treatment	Sulfur Content, %	Ultimate Force, N	Displacement, mm
Muffle Furnace	60	1770.02	3.27
	65	2393.62	3.90
	75	3623.10	3.84
	80	6577.88	3.93
Electric Burner	60	8570.36	1.70
	65	9417.26	1.58
	75	10,002.45	1.58
	80	11,093.49	1.55

## Data Availability

The original contributions presented in this study are included in the article. Further inquiries can be directed to the corresponding author.
